# Critical points of the three-dimensional Bose-Hubbard model from on-site atom number fluctuations

**DOI:** 10.1038/s41598-019-44825-9

**Published:** 2019-06-18

**Authors:** Oskar A. Prośniak, Mateusz Łącki, Bogdan Damski

**Affiliations:** Jagiellonian University, Institute of Physics, Łojasiewicza 11, 30-348 Kraków, Poland

**Keywords:** Ultracold gases, Phase transitions and critical phenomena

## Abstract

We discuss how positions of critical points of the three-dimensional Bose-Hubbard model can be accurately obtained from variance of the on-site atom number operator, which can be experimentally measured. The idea that we explore is that the derivative of the variance, with respect to the parameter driving the transition, has a pronounced maximum close to critical points. We show that Quantum Monte Carlo studies of this maximum lead to precise determination of critical points for the superfluid-Mott insulator transition in systems with mean number of atoms per lattice site equal to one, two, and three. We also extract from such data the correlation-length critical exponent through the finite-size scaling analysis and discuss how the derivative of the variance can be reliably computed from numerical data for the variance. The same conclusions apply to the derivative of the nearest-neighbor correlation function, which can be obtained from routinely measured time-of-flight images.

## Introduction

The field of quantum phase transitions is significant for both fundamental and practical reasons^1–3^. On the fundamental side, it provides insights into rich physics of the most complicated many-body quantum systems whose description challenges the most advanced theoretical and numerical studies. On the practical side, understanding of quantum phase transitions is critical for explanation of properties of numerous materials of potential technological importance such as high-temperature superconductors. One of the key obstacles to progress in the field of quantum phase transitions is our inability to efficiently solve the models describing strongly-correlated systems.

The recent progress in cold atomic physics suggests that it could be possible to approach this problem from another angle. Namely, one can use cold atomic setups as hardware for simulation of condensed matter models. The idea to use an easy-to-control quantum system to simulate another quantum system that is hard-to-study in conventional setups dates back to Feynman^4^. Its proof of principle experimental demonstration was achieved about two decades ago in the cold atom experiment^5^. Ever since this publication, the field of quantum simulation has attracted a lot of attention by providing both a fertile ground for interdisciplinary research and a plausible promise of delivering flexible experimental hardware for solving various challenging models.

At the center of these studies is the implicit assumption that such models can be *quantitatively* studied in cold atom simulators. Despite fascinating experimental efforts that have been reported so far^6,7^, the current characterization of quantum phase transitions in best cold atom simulators is nowhere near the level of accuracy that is found in top condensed matter experiments.

To justify this statement and to place the research discussed in this paper in a broader context, we compare measurements of the lambda transition in liquid ^4^He to measurements of the superfluid–Mott insulator transition in the two-dimensional (2D) cold atom cloud. Such a comparison is meaningful because the two systems are expected to belong to the same universality class, the one of the 3D *XY* model.

**Figure 1:**
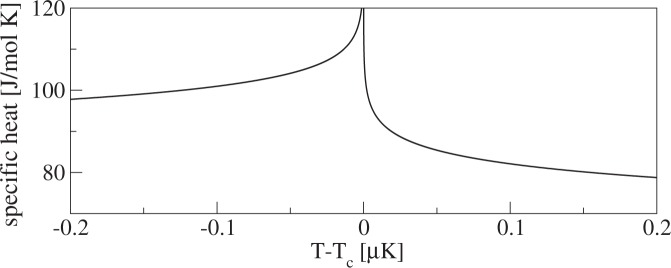
Specific heat of liquid ^4^He near the lambda transition^9^. The curve is given by $A|1-T/{T}_{c}{|}^{-\alpha }+B$, where $\alpha \approx -\,0.0127$, *B* ≈ 460 J/$({\rm{mol}}\cdot {\rm{K}})$, and *T*_*c*_ ≈ 2.17 K. The *A* coefficient for $T < {T}_{c}$ and $T > {T}_{c}$ is approximately given by −447 J/$({\rm{mol}}\cdot {\rm{K}})$ and −471 J/$({\rm{mol}}\cdot {\rm{K}})$, respectively. Its asymmetry produces the lambda shape of the specific heat curve. Specific heat is not divergent at the critical point because the exponent $\alpha  < 0$. Measurements of specific heat as large as 120 J/$({\rm{mol}}\cdot {\rm{K}})$ were reported. A similar shape is observed in our results for ${\partial }_{\eta }{\rm{Var}}$.

The experimental studies of the lambda transition in ^4^He have started about a century ago and continue to this date. The good reason for this long-term interest is that they provide the most stringent tests of the renormalization-group (RG) theory, which is the cornerstone of our understanding of phase transitions. The RG predictions are best established very close to the critical point^8^, where it is difficult to do measurements. One of the key current problems in accurate testing of the RG theory at the lambda transition is that gravity broadens the transition (the transition temperature depends on pressure, which varies across the sample due to the gravitational field). To remove this obstacle, the measurements were taken in a zero-gravity environment in a Space Shuttle^9^. This allowed for checking the RG predictions up to the relative distances $|1-T/{T}_{c}|\approx {10}^{-9}$, where ${T}_{c}\approx 2.17\,{\rm{K}}$ marks the position of the critical point (*T*_*c*_ can be extracted out of the specific heat data from ref.^10^ with relative accuracy of about 10^−8^; ref.^9^ does not provide its value). These measurements, whose main outcome we briefly summarize in Fig. 1, provided the specific heat critical exponent *α* = −0.0127(3). Such a value is close to the exponent that one can theoretically obtain from the studies of the 3D *XY* model^11^. It provides significant evidence about the universality class of the lambda transition. On the less optimistic side, these experiments are quite complicated in execution as they are performed over 200 km above the Earth. It is thus hard to expect that the progress in these studies can be sustained.

These results can be compared to the state of the art experiments in the 2D cold atom clouds placed in a periodic potential formed by standing laser beams (an optical lattice)^12,13^. These experiments aimed at determination of the position of the critical point from the disappearance of the condensate fraction in the Mott insulator phase. They located the critical point with 10–15% relative accuracy, which precluded experimental studies of the cold atom system as near the critical point as in the above-reported measurements in liquid ^4^He. Moreover, the condensate fraction in these experiments was estimated by the relative size of the main peak in time-of-flight images. Such an estimation is heuristic, as the condensate fraction is rather defined as the largest eigenvalue of the single-particle density matrix, which was not studied in refs^12,13^. Finally, these references did not report any measurements of the critical exponents, which excluded experimental identification of the universality class of the studied system.

On the more positive side, recent developments in the experimental manipulations of cold atomic samples suggest that substantial progress in the studies of phase transitions in these systems could be expected. For that purpose, we need efficient tools that can be used to extract quantitative rather than qualitative features of phase transitions out of experimental data. This observation motivates the work presented here.

The purpose of this work is to discuss a practical scheme leading to precise determination of the critical points of the 3D Bose-Hubbard model. In addition to that, the method that we explore provides the correlation-length critical exponent, albeit with lower accuracy. The idea behind these studies was proposed in ref.^14^, where we tested it in the two-dimensional Bose-Hubbard model. The 3D Bose-Hubbard model has been intensively discussed over the past decade because it provides basic description of the properties of cold atoms in optical lattices. Despite all this interest, precision experimental results on its critical properties are hard-to-find in the literature.

The outline of this paper is the following. We will briefly summarize below basic features of the 3D Bose-Hubbard model, state of the art experimental and theoretical results on positions of its critical points, and our scheme for finding them. Then, we will discuss our results coming from Quantum Monte Carlo (QMC) simulations.

## Results

### The model

The Hamiltonian of the 3D Bose-Hubbard model reads^15,16^

1 $$\hat{H}=-\,J\,\sum _{\langle {\bf{i}},{\bf{j}}\rangle }\,({\hat{a}}_{{\bf{i}}}^{\dagger }{\hat{a}}_{{\bf{j}}}+{\hat{a}}_{{\bf{j}}}^{\dagger }{\hat{a}}_{{\bf{i}}})+\frac{U}{2}\,\sum _{{\bf{i}}}\,{\hat{n}}_{{\bf{i}}}({\hat{n}}_{{\bf{i}}}-1),$$

$$[{\hat{a}}_{{\bf{i}}},{\hat{a}}_{{\bf{j}}}^{\dagger }]={\delta }_{{\bf{i}}{\bf{j}}},\,[{\hat{a}}_{{\bf{i}}},{\hat{a}}_{{\bf{j}}}]=0,\,{\hat{n}}_{{\bf{i}}}={\hat{a}}_{{\bf{i}}}^{\dagger }{\hat{a}}_{{\bf{i}}},$$

where $\langle {\bf{i}},{\bf{j}}\rangle$ describes nearest-neighbor sites **i** and **j** in the cubic lattice and periodic boundary conditions are imposed on the creation and annihilation operators. A physical realization of such a model, albeit with open boundary conditions, would require placing cold atoms in a three-dimensional optical lattice enclosed in an optical box trap. The tools needed for experimental creation of such a trap have been recently developed^17–22^.

A comprehensive review of the properties of this model can be found in ref.^23^. In short, its physics depends on the filling factor *n*, i.e. the mean number of atoms per lattice site, and the ratio of the tunneling coupling *J* to the interaction energy *U*

2 $$\eta =J/U.$$

We are interested in integer filling factors, for which there is a quantum phase transition^15^ between the Mott insulator and the superfluid phase at the critical point ${\eta }_{c}={J}_{c}$/${U}_{c}$. The system is in the Mott insulator phase for $\eta  < {\eta }_{c}$ and in the superfluid phase for $\eta  > {\eta }_{c}$.

The critical point at unit filling factor was theoretically studied via perturbative expansions^24,25^, QMC simulations^26^, the non-perturbative renormalization group approach^27^, and the projection operator approach^28^. The critical points at higher filling factors were studied perturbatively in ref.^24^ for $n=2,3$ and in ref.^25^ for an arbitrary filling factor. The results of all above-mentioned papers can be summarized as follows

3 $${\eta }_{c}\approx \{\begin{array}{ll}0.034 & {\rm{for}}\,n=1\\ 0.02 & {\rm{for}}\,n=2\\ 0.014 & {\rm{for}}\,n=3\end{array}.$$

We will now summarize experimental results on the critical points of the 3D Bose-Hubbard model^5,29,30^. The experiment^5,31^ is done in an optical lattice of wavelength $\lambda =852\,{\rm{nm}}$ with ^87^Rb atoms in the $|F=2,{m}_{F}=2\rangle$ state. Their s-wave scattering length^32^ is 5.45(26) nm. The position of the critical point for the unit filling factor was estimated to correspond to lattice heights between 10*E*_*R*_ and 13*E*_*R*_, where the recoil energy *E*_*R*_ is defined as ${\hslash }^{2}{k}^{2}$/2*m* with $k=2\pi$/*λ* and *m* being the mass of the considered atom. This may be written as 11.5(9)*E*_*R*_, where the standard deviation has been estimated^33^ by dividing maximum uncertainty of 1.5*E*_*R*_ by $\sqrt{3}$. Using the formulas for *J* and *U* coefficients from^23^, we find ${\eta }_{c}$ to be 0.04(1). The number reported in the bracket provides one standard deviation due to uncertainties in the lattice height and scattering length. It has been obtained through the uncertainty propagation formula. Nearly identical experimental setup was used in^29^. It was found there that the lattice heights corresponding to critical points for double and triple filling factors are 14.1(8)*E*_*R*_ and 16.6(9)*E*_*R*_, respectively. Applying the same procedure as above, albeit with *λ* = 850 nm, we get ${\eta }_{c}$ for the double and triple filling factors 0.021(5) and 0.011(2), respectively. Finally, we come to ref. ^30^, where again ^87^Rb atoms, but in a different hyperfine state, are studied. It was found there that the superfluid–Mott insulator transition takes place at ${\eta }_{c}=0.029(2)$ for the unit filling factor.

To put these results in perspective, we can compare them to the mean-field predictions, which for our system are^34^

4 $${\eta }_{c}=\frac{2n+1-2\sqrt{n(n+1)}}{6}.$$

This yields ${\eta }_{c}$ equal to 0.029, 0.017, and 0.012 for *n* = 1, 2, and 3, respectively. Therefore, we see that more accurate experimental results are needed for characterization of beyond mean-field effects in the position of the critical points. It should be also said that in all above-mentioned experiments external harmonic trapping is imposed on the system. At the very least, it enhances finite-size effects making detailed comparision between experiments and the theory based on Hamiltonian (1) difficult. Such comparision is additionally complicated by the fact that the 3D Bose-Hubbard model captures only the leading-order behavior of cold atoms in optical lattices^35^. As a result, more precise experimental results on the critical points would presumably ask for a bit more advanced theoretical description of the system. Our method for locating the critical points should be immediately applicable to such non-standard versions of the 3D Bose-Hubbard model.

Besides critical points, quantum phase transitions are also characterized by critical exponents, which are supposed to be the same within a given universality class. The quantum 3D Bose-Hubbard model belongs to the universality class of the classical 4D *XY* model^15^. To the best of our knowledge, however, detailed studies of the critical properties of the latter model have not been presented in the literature so far. This is in sharp contrast to the properties of the lower-dimensional *XY* models, which have been studied in great detail^36^. The difficulties presumably arise here from the complexity of numerical studies of such a high-dimensional model.

Furthermore, we note that the upper critical dimension of the *XY* model is four. This means that the mean-field theory, whose dynamical *z* and correlation-length *ν* critical exponents are given by

5 $$z=1,\,\nu =1/2,$$

should provide the lowest-order approximation to behavior of the 4D *XY* model. As it will turn out below, our relatively small system-size simulations are unable to capture corrections to the mean-field values of the critical exponents.

### The observable of interest

We will study here variance of the on-site atom number distribution

6 $${\rm{Var}}=\langle {\hat{n}}_{{\bf{i}}}^{2}\rangle -{\langle {\hat{n}}_{{\bf{i}}}\rangle }^{2},$$

where the site index **i** can be chosen arbitrarily due to the translational invariance of our model. Such an observable can be conveniently computed with QMC algorithms. It can be also experimentally measured^37–39^. Alternatively, since we are actually interested in the derivative of the variance, one may focus on the derivative of the nearest-neighbor correlation function and use the mapping

7 $$\frac{\partial }{\partial \eta }{\rm{Var}}=6\eta \frac{\partial }{\partial \eta }C,\,C={\langle {\hat{a}}_{{\bf{i}}}^{\dagger }{\hat{a}}_{{\bf{j}}}+{a}_{{\bf{j}}}^{\dagger }{\hat{a}}_{{\bf{i}}}\rangle |}_{\langle {\bf{i}},{\bf{j}}\rangle },$$

which can be obtained through the Feynman-Hellmann theorem (it is strictly valid in systems that are either thermodynamically-large or periodic). The nearest-neighbor correlation function can be extracted out of the routinely measured time-of-flight images in cold atom simulators^7,38^.

The variance can be perturbatively calculated deeply in the Mott insulator phase at temperature of absolute zero^23^

8 $$\langle {\hat{n}}_{{\bf{i}}}^{2}\rangle -{\langle {\hat{n}}_{{\bf{i}}}\rangle }^{2}\approx \{\begin{array}{ll}24{\eta }^{2}+3960{\eta }^{4} & {\rm{for}}\,n=1\\ 72{\eta }^{2}+33408{\eta }^{4} & {\rm{for}}\,n=2\\ 144{\eta }^{2}+131400{\eta }^{4} & {\rm{for}}\,n=3\end{array}.$$

The higher-order zero-temperature perturbative calculations of the variance in the Mott insulator phase of the 3D Bose-Hubbard model were numerically performed in comprehensive work of Teichmann *et al*.^25^.

### Quantum Monte Carlo simulations

We perform QMC simulations, which we briefly describe in the Methods section (see^40^ for a cold-atom-oriented review of this subject). This allows us to study physics on the superfluid side of the transition, where dependence of the variance on $\eta$ is most interesting for our purposes. Additionally, this approach allows us to get nonzero-temperature results, which is of interest from the experimental perspective.

We perform our studies in lattices of size

9 $${L}^{3},$$

where the linear system size $4\le L\le 16$ for $n=1,2$ and $4\le L\le 12$ for $n=3$. Most of the time, we investigate systems at temperature ${k}_{B}T$/$U=0.02$, where *k*_*B*_ is the Boltzmann constant. Such temperature can be converted to Kelvins by considering typical experimental conditions. To do so, one may assume that the lattice of wavelength *λ* = 532 nm is populated by either ^87^Rb or ^174^Yb atoms having the s-wave scattering lengths of 5.45 nm and 5.56 nm, respectively^32,41^. Using then formulas from^23^, we find that *U*_*c*_/*E*_*R*_ for $n=1,2,3$ equals 0.47, 0.54, 0.59 for ^87^Rb and 0.48, 0.55, 0.60 for ^174^Yb. These numbers have been obtained by assuming that critical points are given by (3). Since we work near critical points, we may take *U*_*c*_/*k*_*B*_ as the unit of temperature. In nano Kelvins, it equals 184, 211, 230 (93, 107, 117) for $n=1,2,3$ in ^87^Rb (^174^Yb). We have checked that the same results can be obtained by generating Wannier functions and then integrating them over in order to get *J* and *U* coefficients^16^.

We see from these calculations that temperature ${k}_{B}T$/$U=0.02$ corresponds to a few nano Kelvins in typical rubidium and ytterbium systems. While such low temperatures are certainly experimentally challenging, it does not mean that our studies are completely free from nonzero-temperature corrections. For example, the 3D Bose-Hubbard model was studied through QMC simulations in ref.^26^ at temperature about twenty times smaller than our ${k}_{B}T$/$U=0.02$. These studies were done at the unit filling factor in systems, whose sizes were similar to the ones used by us. The critical point was extracted from finite-size scaling of the excitation gap. The relative difference between the position of the critical point found in our work and the one from ref.^26^ is about 0.5%. We will thus skip systematic discussion of nonzero-temperature effects in our computations. It should be stressed, however, that our approach to finding critical points can be applied to “warmer” systems as well, where nonzero-temperature scaling analysis can be deployed^3^.

**Figure 2:**
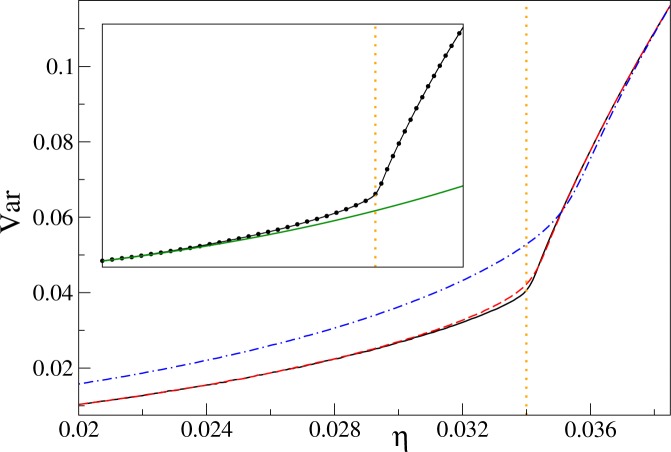
The variance of the on-site number operator at the unit filling factor. Main plot: curves show QMC data points connected by lines for ${k}_{B}T$/*U* equal to 0.02 (solid black), 0.04 (dashed red), and 0.08 (dashed-dotted blue). Inset: the circles are QMC data from the main plot for ${k}_{B}T$/$U=0.02$, the solid black line is the Padé approximant fitted to the numerics, and the green solid line is perturbative result (8) obtained for ${k}_{B}T$/$U=0$. The range of the axes in the inset is the same as in the main plot. Both here and in the following plots the vertical dotted orange lines mark the position of the critical point (3). The linear system size is $L=16$.

**Figure 3:**
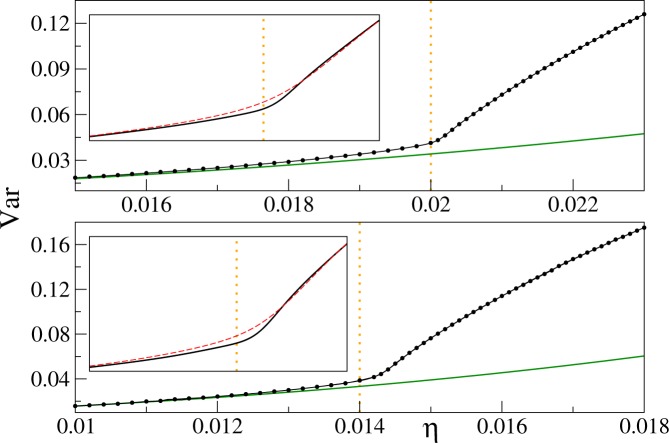
The variance of the on-site atom number operator at filling factors $n=2$ (upper panel) and 3 (lower panel). Upper panel: the solid black line shows the Padé approximant for the linear system size $L=16$. The inset illustrates differences between Padé approximants for $L=16$ (black line) and $L=8$ (red dashed line) near the critical point. The range of the horizontal (vertical) axis in the inset is $0.0185\le \eta \le 0.021$ ($0.03\le {\rm{Var}}\le 0.075$). Lower panel: the solid black line depicts the Padé approximant for $L=12$. The inset shows differences around the critical point between Padé approximants for $L=12$ (black line) and $L=6$ (red dashed line). The range of the horizontal (vertical) axis in the inset is $0.012\le \eta \le 0.0155$ ($0.022\le {\rm{Var}}\le 0.1$). The solid green line in both panels shows perturbative results (8) for ${k}_{B}T$/$U=0$. All other results, including circles showing QMC data, are obtained for ${k}_{B}T$/$U=0.02$.

**Figure 4:**
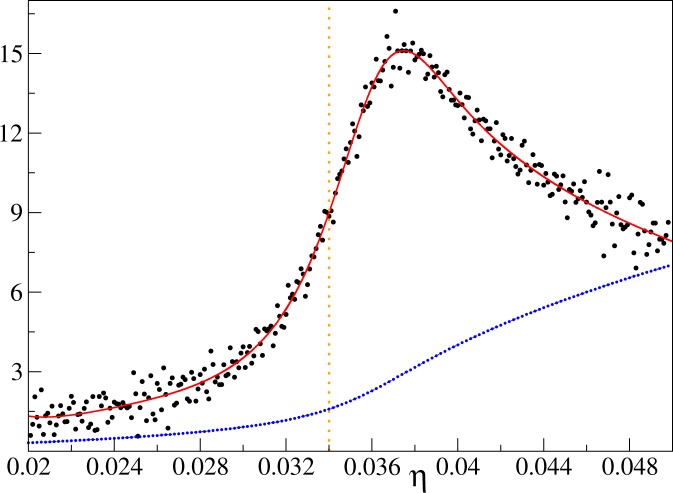
The first derivative of the variance for the unit filling factor: raw data vs. the Padé approximant result. Black dots: the central difference numerical derivative of the QMC data for the variance. Three hundred equally-spaced data points were used to compute the derivative. The value represented by every other such data point is multiplied by a factor of thirty and depicted as a small blue dot in the plot. The solid red line is the derivative of the Padé approximant that has been fitted to the QMC numerics. Computations for this plot have been done for ${k}_{B}T$/$U=0.02$ and the linear system size $L=4$.

The variance for the filling factors *n* = 1 and *n* = 2, 3 is presented in Figs 2 and 3, respectively. We see there its steep increase around the expected positions of critical points (3). To locate the points, where changes of the variance proceed most rapidly, we compute the first derivative of the variance. Such a procedure encounters a technical problem: the derivative is sensitive to fluctuations of data that is being differentiated (Fig. 4). Therefore, such data has to be smoothed first, which we do by fitting the Padé approximant of order (*m*, *s*)^42^

10 $${\rm{Var}}(\eta )=\frac{{\sum }_{i=0}^{m}\,{A}_{i}{\eta }^{i}}{1+{\sum }_{i=1}^{s}\,{B}_{i}{\eta }^{i}}.$$

Usefulness of this procedure is illustrated in Fig. 4, where we see that the derivative of the Padé approximant provides a smooth curve that can be easily subjected to detailed analysis. We mention in passing that the very same procedure could be applied to data for the variance coming from experimental measurements.

Fitting noisy data with (10) requires the approximation order to be adapted to the problem. While small orders may result in a bad fit due to insufficient number of fitting parameters, choosing too large orders causes problems as well. In the latter case, such extra flexibility leads to reproduction of noise-induced fluctuations of data points instead of averaging the fluctuations out.

**Figure 5:**
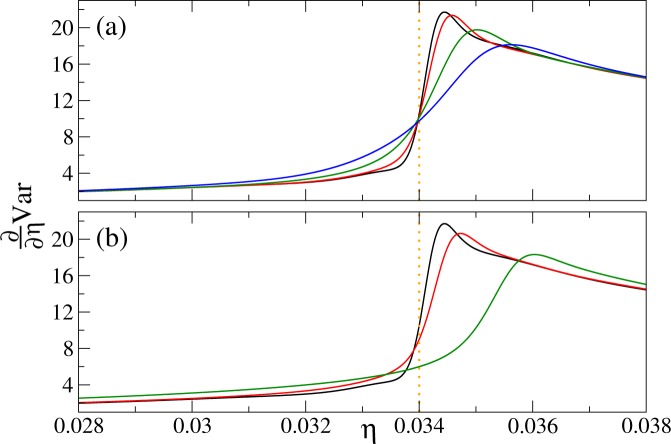
The first derivative of the variance of the on-site atom number operator at the unit filling factor. The derivative is computed from Padé approximants to QMC data. Panel (a) system-size dependence for ${k}_{B}T$/$U=0.02$. The curves from top to bottom correspond to linear system sizes *L* equal to 16 (black), 12 (red), 8 (green), and 6 (blue), respectively. Panel (b) temperature dependence for $L=16$. The curves from top to bottom correspond to *k*_*B*_*T*/*U* equal to 0.02 (black), 0.04 (red), and 0.08 (green), respectively.

**Figure 6:**
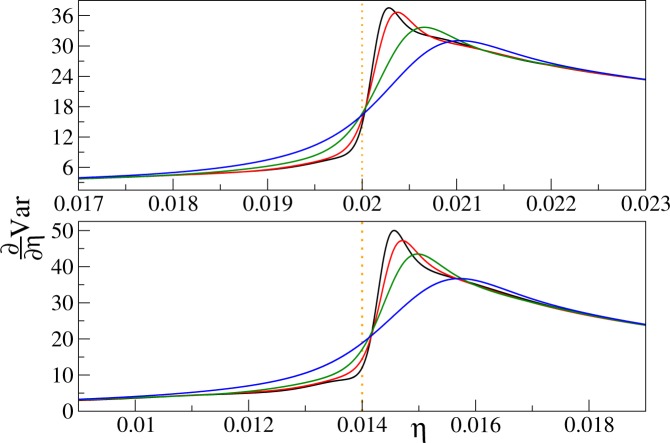
The first derivative of the variance of the on-site atom number operator at filling factors *n* = 2 (upper panel) and 3 (lower panel). Upper panel: the curves from top to bottom are for linear system sizes *L* equal to 16 (black), 12 (red), 8 (green), and 6 (blue). Lower panel: the curves from top to bottom are prepared for *L* equal to 12 (black), 8 (red), 6 (green), and 4 (blue). Derivatives of Padé approximants are displayed in both panels, all calculations are done for *k*_*B*_*T*/*U* = 0.02.

**Figure 7:**
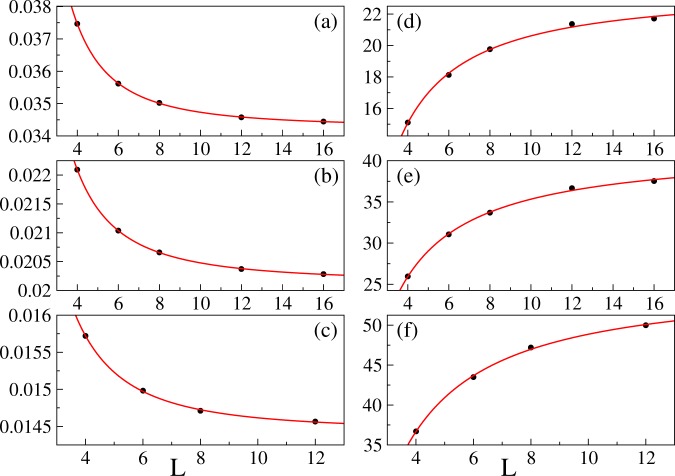
Properties of the maximum of the first derivative of the variance. The black dots are extracted from Padé approximants to QMC data, the red solid lines are nonlinear fits (11) and (18). Panels (a–c) show ${\eta }^{\ast }$ for filling factors *n* = 1, 2, and 3, respectively. The fitted curves are: $0.03427(2)+0.060(3){L}^{-2.12(4)}$, $0.020144(8)+0.0282(8){L}^{-1.93(2)}$, and $0.01444(4)+0.026(6){L}^{-2.2(2)}$ (top to bottom). Panels (d–f) show ${\partial }_{\eta }{\rm{Var}}({\eta }^{\ast })$ for filling factors *n* = 1, 2, and 3, respectively. The fitted curves are: 23.6(7) − 42(8)*L*^−1.2(2)^, 42(1) − 62(8)*L*^−1.0(1)^, and 55(2) − 94(18)*L*^−1.2(2)^ (top to bottom). We report here one standard deviation in the brackets next to the fitted coefficients. The fitting is done with NonlinearModelFit function from^52^. All results are for *k*_*B*_*T*/*U* = 0.02.

**Figure 8:**
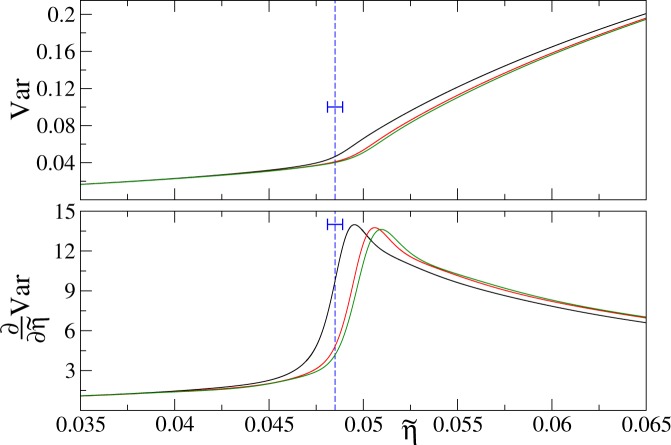
The variance of the on-site atom number operator and its derivative as a function of rescaled variable (23). The solid black, red, and green lines–top to bottom in the upper panel and left to right in the lower panel–present Padé approximants to our QMC numerics for filling factors *n* = 1, 2, and 3, respectively. The critical values of $\tilde{\eta }$, which can be obtained from (3), are approximately 0.0481, 0.0490 and 0.0485 for filling factors *n* = 1, 2 and 3, respectively. The vertical dashed blue lines are placed at $\tilde{\eta }$ equal to 0.0485, the mean of the above-reported numbers. The spread of the positions of the critical points for different filling factors around such mean, ±0.0004, is marked by the horizontal “error bars”. All results are for the linear system size *L* = 8 and temperature *k*_*B*_*T*/*U* = 0.02.

Choosing the optimal order of the Padé approximant is not difficult in our computations. Indeed, we have found that for every combination of (*L*, *n*, *T*) parameters, there exists a stability island in the set of all reasonable approximation orders. By taking the order of approximation within the island, stable results for the variance and its derivative are obtained. We have found that typically our QMC data sets can be reasonably fitted with $8\le m$, $s\le 9$. We have also found that by considering a denser numerical grid, or by reducing the QMC noise by increasing the sample size, stable results can be obtained. We have applied this strategy for creation of Figs 2–8, where Padé approximants of the fixed (8, 8) order are employed.

The system-size and temperature dependence of ${\partial }_{\eta }{\rm{Var}}$ is presented in Figs 5 and 6 for the filling factors $n=1$ and $n=2,3$, respectively. The first thing we notice there is the lambda-shape of the plots reminiscent of the specific-heat plot in liquid ^4^He (Fig. 1).

Then, we find that the first derivative of the variance has a maximum near the critical point on the superfluid side of the transition, say at ${\eta }^{\ast }$. We see that ${\eta }^{\ast }$ shifts towards the critical point as the system size increases. The same is observed when temperature decreases. Moreover, ${\partial }_{\eta }{\rm{Var}}({\eta }^{\ast })$ grows with the system size and inverse temperature. All these observations suggest that the maximum of the derivative of the variance encodes the position of the critical point. This is not the first time when the derivative of an experimentally-accessible quantity is used for finding the critical point of the 3D Bose-Hubbard model. Indeed, the derivative of experimentally-measured visibility of the time-of-flight interference pattern was used for such a purpose as well^29^.

More quantitatively, we study the position of the maximum of ${\partial }_{\eta }{\rm{Var}}$ by fitting

11 $$A+B{L}^{-C}$$

to numerical results. The idea here is that the parameter *a* estimates the position of the maximum in the thermodynamically-large system ($c > 0$). The typical fits that we perform are shown in Fig. 7a–c, where the positions of the maxima have been extracted from Padé approximants of order (8, 8). To check sensitivity of these results to the order of approximants (10), we have done calculations for

12 $$7\le m,s\le 9.$$

We have obtained

13 $$a=\{\begin{array}{ll}0.03430\pm 0.00008 & {\rm{for}}\,n=1\\ 0.02020\pm 0.00006 & {\rm{for}}\,n=2\\ 0.01447\pm 0.00003 & {\rm{for}}\,n=3\end{array},$$

where the error bars are chosen to capture all the results in the parameter range given by (12). Standard deviations of fitted coefficients for $m=s=8$ are typically a bit smaller (Fig. 7). A quick look at (3) reveals that these results provide positions of critical points, which makes us confident that ${\eta }^{\ast }$ converges to ${\eta }_{c}$ in the thermodynamic limit.

For the *c* coefficient under constraint (12), we have got

14 $$c=\{\begin{array}{ll}2.11\pm 0.08 & {\rm{for}}\,n=1\\ 1.97\pm 0.08 & {\rm{for}}\,n=2\\ 2.23\pm 0.05 & {\rm{for}}\,n=3\end{array},$$

which can be explained through the standard finite-size scaling argument^8,43^. Namely, we assume that near the critical point the system properties depend on the linear system size and the ratio between the linear system size and the correlation length $\xi$, say

15 $${\partial }_{\eta }{\rm{Var}}=h(L)f(\frac{L}{\xi }),\,\xi \sim |\eta -{\eta }_{c}{|}^{-\nu },$$

where *f* is a non-singular scaling function. According to this ansatz, the position of the extremum of ${\partial }_{\eta }{\rm{Var}}$ scales

16 $$|{\eta }^{\ast }-{\eta }_{c}|\sim {L}^{-1/\nu }$$

and we see that the function *h*(*L*) captures system-size dependence of ${\partial }_{\eta }{\rm{Var}}$ at ${\eta }^{\ast }$. The exponent in this equation matches the *c* parameter if we use the mean-field value of the critical exponent *ν* (5). More accurate studies are needed for checking if there are beyond-mean-field corrections to the critical exponent *ν* in the 3D Bose-Hubbard model. We believe that the key limitations here come from the relatively small system sizes that can be numerically handled.

Finally, for the sake of completeness, we provide the results for the fitting parameter *b* again under the variation of the order of Padé approximation (12)

17 $$b=\{\begin{array}{ll}0.059\pm 0.005 & {\rm{for}}\,n=1\\ 0.029\pm 0.002 & {\rm{for}}\,n=2\\ 0.028\pm 0.002 & {\rm{for}}\,n=3\end{array}.$$

Next, we will discuss scaling of ${\partial }_{\eta }{\rm{Var}}({\eta }^{\ast })$ with the linear system size. We fit

18 $$A+B{L}^{-C}$$

to numerics. Typical results that we obtain are presented in Fig. 7d–f, where again Padé approximants of order (8, 8) have been employed. Next, we quantify influence of the order of Padé approximation on these results. Proceeding similarly as with (13), (14) and (17), we get the results summarized in Table 1. All of them suggest that ${\partial }_{\eta }{\rm{Var}}({\eta }^{\ast })$ slowly increases with the linear system size reaching a finite value in the thermodynamic limit. This has an interesting consequence that can be readily spotted in Figs 5 and 6.

**Table 1:** Coefficients obtained by fitting (18) to ∂_*η*_Var(η*).

	A	B	C
*n* = 1	23.3 ± 0.6	−44 ± 4	1.2 ± 0.1
*n* = 2	41 ± 1	−64 ± 4	1.1 ± 0.1
*n* = 3	54 ± 1	−101 ± 7	1.3 ± 0.1

Error bars capture spread of the results due to the varying order of Padé approximation (12). QMC results for *k*_*B*_*T*/*U* = 0.02 have been used to prepare this table, the same system sizes as in Fig. 7 have been employed in the fitting.

Namely, we see that the curves showing ${\partial }_{\eta }{\rm{Var}}$ for different system sizes at constant temperature cross near the critical point. This can be explained by the finite-size scaling ansatz (15), if we note that ${\partial }_{\eta }{\rm{Var}}({\eta }_{c})\sim h(L)$ and take into account that *h*(*L*) weakly depends on the linear system size reaching a finite value in the thermodynamic limit. The latter remark follows from the fact that *h*(*L*) is proportional to ${\partial }_{\eta }{\rm{Var}}({\eta }^{\ast })$, which we have just discussed. We mention in passing that similar-looking crossing of curves near the critical point was used for finding the position of the critical point from QMC data for the excitation gap^26^.

Further insights into ${\partial }_{\eta }{\rm{Var}}$ can be obtained by setting ${k}_{B}T$/$U=0$ and using the Feynman-Hellmann theorem to arrive at^14^

19 $$\frac{\partial }{\partial \eta }{\rm{Var}}=-\,2\eta \frac{{\partial }^{2}}{\partial {\eta }^{2}}(\frac{ {\mathcal E} }{U}),$$

where ${\mathcal E}$ is the ground-state energy per lattice site. This expression is closely linked to the one for specific heat oftentimes studied in the context of classical phase transitions (see e.g. Fig. 1 and the discussion around it). Indeed, specific heat per lattice site can be written as^44^

20 $$-\,T\frac{{\partial }^{2}}{\partial {T}^{2}} {\mathcal F} ,$$

where ${\mathcal F}$ is the free energy per lattice site. Its singular part is typically assumed to scale as $|T-{T}_{c}{|}^{2-\alpha }$, where *α* is the specific heat critical exponent. A quick look at (19) and (20) reveals the mapping between the two expressions. It is then unsurprising that the singular part of ${\mathcal E}$ is usually assumed to scale as

21 $$|\eta -{\eta }_{c}{|}^{2-\alpha }.$$

The exponent *α* is linked to the *z* and *ν* critical exponents through the quantum hyperscaling relation

22 $$\alpha =2-\nu (d+z),$$

where *d* is the system’s dimensionality^3^.

Combining (19) with (21), one gets ${\partial }_{\eta }{\rm{Var}}\sim |\eta -{\eta }_{c}{|}^{-\alpha }$ in the infinite system, which would imply that $h(L)\sim {L}^{\alpha /\nu }$. As a result, $\alpha =0$ would be compatible with our numerics in the large-*L* limit. Such a value can be obtained by putting mean-field critical exponents (5) into (22). There are, however, at least two reasons to be cautious here.

First, the upper critical dimension of the Bose-Hubbard model is three and so it is expected that there will be corrections to the mean-field scaling laws. As a result, it is unclear to us what is the actual value of *α*.

Second, even if *α* would be zero, the presence of logarithmic singularities in the derivatives of the ground-state energy could not be ruled out without detailed analysis. For example, such a situation takes place in the one-dimensional quantum Ising model, where $\alpha =0$ due to $z=\nu =d=1$^45,46^. The singular part of its ground-state energy per lattice site ${ {\mathcal E} }_{{\rm{Ising}}}$ turns out to be proportional to ${(g-{g}_{c})}^{2}\,\mathrm{ln}\,|g-{g}_{c}|$ in the thermodynamically-large chain, where *g* is the magnetic field driving the transition and *g*_*c*_ is the critical point. As a result, ${\partial }_{g}^{2}{ {\mathcal E} }_{{\rm{Ising}}}$ diverges logarithmically with $|g-{g}_{c}|$. In the finite chain, there is the extremum of ${\partial }_{g}^{2}{ {\mathcal E} }_{{\rm{Ising}}}$ at say g*. When the system size increases, ${g}^{\ast }\to {g}_{c}$ and $|{\partial }_{g}^{2}{ {\mathcal E} }_{{\rm{Ising}}}({g}^{\ast })|\to \infty$. The latter property differs from what we seem to observe in the 3D Bose-Hubbard model.

One possible explanation of our puzzling observation that ${\partial }_{\eta }{\rm{Var}}({\eta }^{\ast })$ reaches a finite value in the thermodynamic limit might be that the system sizes that we consider are much too limited rendering our extrapolations unreliable. Still, the use of our fitting results for interpolation purposes should be very well justified and useful.

Finally, we would like to briefly discuss dependence of our results on the filling factor *n*. The idea that we explore here comes from Teichmann *et al*.^25^, where it was found through perturbative studies that deeply in the Mott insulator phase the variance of the on-site atom number operator is a function of

23 $$\tilde{\eta }=\sqrt{n(n+1)}\eta .$$

This implies that ${\partial }_{\tilde{\eta }}{\rm{Var}}$ should be a function of (23) as well.

Our QMC numerics, which we present in Fig. 8, perfectly follows these predictions away from the critical point on the Mott insulator side of the transition. We also see in this figure that the mapping $\eta \mapsto \tilde{\eta }$ fails a bit in the superfluid phase for low-*n* data that we explore. Nonetheless, judging from quite good overlap between the *n* = 2 and 3 results, it is reasonable to expect that the mapping will be accurately supported by numerical simulations in both phases in the limit of $n\gg 1$. Further studies are needed for establishing this observation.

## Discussion

We have studied equilibrium properties of the 3D Bose-Hubbard model focusing our attention on the variance of the on-site atom number operator and its derivative with respect to the parameter driving the superfluid-Mott insulator transition. Our results have been obtained in systems with the mean number of atoms per lattice site equal to one, two, and three. They come from Quantum Monte Carlo simulations.

The key finding of this work is that the derivative of the variance has a pronounced maximum close to critical points. For example, in a very small lattice of linear size 4, when the number of atoms equals the number of lattice sites, the position of the maximum estimates the position of the critical point with 10% relative accuracy (Fig. 4). The mismatch between the two decreases quadratically with the linear system size and it can be further suppressed by simple extrapolation to the thermodynamic limit.

Besides discussing the position of the critical point, which is an interesting albeit non-universal feature, we have found that even in small systems the critical exponent *ν* can be extracted from the finite-size shift of the maximum of the derivative of the variance. This is interesting because knowledge of this exponent can provide important information about the universality class of the 4D *XY* model.

This is the least-studied universality class of the *XY* model. Limited knowledge of its properties stems from numerical shortcomings, clearly seen in our work, and difficulties in finding physical systems, where it can be experimentally approached. The latter can be done in condensed matter and atomic physics setups. In the condensed matter context, it was proposed that some properties of either strongly underdoped cuprate superconductors or ^4^He in nanoporous media can be captured by the 4D *XY* universality class^47–49^. In the atomic physics context, cold atoms in a three-dimensional optical lattice are the best example of a system whose scaling properties should mirror those of the 4D *XY* model.

We view cold atom setups, simulating the 3D Bose-Hubbard model, as the cleanest and most promising platform for future quantitative studies of the 4D *XY* universality class. In fact, measurements of the on-site atom number fluctuations in the 3D Bose-Hubbard systems have been recently reported^38,39^. Direct comparision of these results to our findings is difficult because setups studied in^38,39^ are non-uniform due to the external trapping potential adding a local chemical-potential-like term to Hamiltonian (1). We are hopeful, however, that blending of techniques presented in these references with the recent optical box trapping advances can lead to successful creation of the homogeneous 3D Bose-Hubbard quantum simulator. Such a system could be large-enough to overcome small-size limitations plaguing numerical simulations. As a matter of fact, quantum simulators, at the very least, are supposed to do just that.

## Methods

We use the Directed Worm Algorithm from the ALPS software package^50,51^. This algorithm samples the path-integral representation of a density matrix of a grand canonical ensemble (GCE) with configurations called worldlines. Since we work with systems having fixed filling factor *n*, computation of any average in a lattice of the linear size *L* requires rejecting those worldlines, where the total numer of particles differs from *nL*^3^. To improve the sample count of the remaining fraction, the chemical potential is adjusted to set the expected GCE density of the system to *n* particles per site. The statistical error of the determined variance is significantly reduced by adopting periodic boundary conditions, where observables do not depend on the lattice site. As a result, the variance of the on-site atom number operator can be averaged over the resulting ensemble and over all *L*^3^ lattice sites, which is exactly what we do.

Due to the amount of computational power needed, our QMC simulations are limited to system sizes and temperatures discussed below equation (9). Early symptoms of these limitations can be spotted in Figs 5 and 6. We see there that for the largest systems considered, there is a small warp in the derivative of the variance slightly to the left of the dotted lines marking positions of critical points. Therefore, it is important to check that positions of the maxima, which we extensively study, are stable under change of parameters of our QMC simulations. Several tests are thus performed. First, we vary the total number of later averaged worldlines reaching typically the level of 10^7^ to 10^8^. Second, when generating the worldlines, only every *m*-th trajectory is included into the final ensemble (if it additionally contains *nL*^3^ particles), to ensure that subsequent worldlines in the ensemble are independent from each other. We check sensitivity of our results to this so-called skip *m* parameter by changing it from 64 to 256. Third, we fit Padé approximants to data sets that differ from each other by range and grid density of the parameter *η*. All these tests make us confident that the results that we present for the maxima of the derivative of the variance are well-converged.
